# Locomotor circumvention strategies are altered by stroke: I. Obstacle clearance

**DOI:** 10.1186/s12984-017-0264-8

**Published:** 2017-06-15

**Authors:** Anuja Darekar, Anouk Lamontagne, Joyce Fung

**Affiliations:** 1 School of Physical and Occupational Therapy, Faculty of Medicine, McGill University, Montreal, Quebec, Canada; 2 Feil and Oberfeld Research Center, Jewish Rehabilitation Hospital of the Centre Intégré de Santé et Services Sociaux de Laval (CISSS-Laval), Research site of the Montreal Centre for Interdisciplinary Research in Rehabilitation (CRIR), Laval, Quebec, Canada

**Keywords:** Collision avoidance, Gait, Navigation, Virtual reality, Stroke, Rehabilitation

## Abstract

**Background:**

Functional locomotion requires the ability to adapt to environmental challenges such as the presence of stationary or moving obstacles. Difficulties in obstacle circumvention often lead to restricted community ambulation in individuals with stroke. The objective of this study was to contrast obstacle circumvention strategies between post-stroke (*n* = 12) and healthy individuals (*n* = 12) performing locomotor and perceptuomotor (joystick navigation) tasks with different obstacle approaches.

**Methods:**

Participants walked and navigated with a joystick towards a central target, in a virtual environment simulating a large room, while avoiding an obstacle that either remained stationary at the pre-determined point of intersection or moved from head-on or diagonally 30° left/right. The outcome measures included dynamic clearance (DC), instantaneous distance from obstacle at crossing (IDC), number of collisions and preferred side of circumvention. These measures were compared between groups (stroke vs. healthy), obstacle parameter (stationary vs. moving head-on) and direction of approach (left/paretic vs. right/non-paretic).

**Results:**

DC was significantly larger when circumventing a moving obstacle that approached head-on as compared to a stationary obstacle for both groups during both tasks, while not significantly different in either diagonal approach in either group. IDC was smaller in the stroke group while walking and larger in both groups during joystick navigation when avoiding moving as compared to stationary obstacle. IDC was significantly larger in the stroke group compared to controls for diagonal approaches during walking, wherein two different strategies emerged amongst individuals with stroke: circumventing to the same (V_same_ *n* = 6) or opposite (V_opp_ *n* = 4) side of obstacle approach. This behavior was not seen in the perceptuomotor task, wherein post-stroke participants circumvented to opposite side of the obstacle approach as seen in healthy participants. In the locomotor task, the V_same_ subgroup that had greater functional limitations used larger DC as compared to the V_opp_ subgroup and healthy individuals. The remaining two individuals with stroke collided with obstacles in >50% trials of either obstacle approach. The underlying mechanisms for collision were however different for both individuals.

**Conclusion:**

Avoidance strategies in individuals with stroke can vary depending on the individual locomotor capabilities and obstacle characteristics.

## Background

The majority of community-dwelling stroke survivors (~50–74%) have restrictions in community participation [1, 2] that could be related to limitations in ambulation [3–5]. Community environments are complex and present substantial challenges to mobility, which need to be overcome with effective locomotor adaptations [6, 7]. Many stroke survivors are known to have gait dysfunctions [8–10] that may limit their locomotor adaptability to perform complex tasks during community ambulation [11–16]. Stationary and moving obstacles are encountered frequently in the community [4, 17] and stroke survivors risk collisions with them or simply avoid going out to crowded environments altogether [4]. Despite this, obstacle circumvention is not routinely assessed or trained during rehabilitation in the post-stroke population [7].

While obstacle circumvention has been investigated in other populations [18–24], there is limited understanding of the mechanisms and strategies used by individuals with stroke in comparison with healthy individuals when circumventing both stationary and moving obstacles. Further, the dynamic interaction between an individual and obstacle is reflected in the dynamic clearance from the obstacle during the avoidance strategy. We have created a unique paradigm using virtual environment and obstacles to assess obstacle circumvention in a previous exploratory study [25], and discovered that post-stroke individuals used cautious strategies (by maintaining larger clearances) when encountered with moving obstacles and that these strategies were influenced by obstacle approach direction. However, the study was limited by a small sample, while stationary obstacles were not used, precluding the contrast with added sensory processing imposed by moving obstacles.

Circumvention strategies can differ between obstacle approach directions such that successful avoidance can be achieved with path adjustments only for obstacles approaching head-on as opposed to changing path and/or speed for diagonally approaching obstacles [26, 27]. Moreover, when steering in a new direction, a difference in coordination strategies was observed when turning to the paretic as opposed to the non-paretic side in individuals with stroke [16]. Likewise, since circumvention frequently involves a transient change in walking direction, different avoidance strategies may be used to avoid obstacles approaching from the paretic or non-paretic side. Further, a seated perceptuomotor joystick navigation task can be used to investigate the cognitive-perceptual and planning aspects of the circumvention task, by minimizing the biomechanical and motor constraints imposed by stroke on walking [25].

The objectives of this study were to compare obstacle circumvention strategies between post-stroke and healthy individuals when circumventing (1) stationary vs. moving obstacles and (2) obstacles approaching diagonally at 30° from the paretic (P) vs. non-paretic (NP) side in a locomotor and a perceptuomotor task. We hypothesized that the stroke group would make larger clearance from the obstacle as compared to the healthy individuals, and when the obstacle is stationary as compared to moving [19]. We further hypothesized that avoidance behavior would be different for obstacles approaching from the paretic compared to non-paretic side during walking but not in the perceptuomotor task of joystick navigation wherein avoidance behavior would be similar to controls across obstacle contexts.

## Methods

### Participants

Twelve chronic stroke and twelve healthy control participants were included in this study. This sample size was selected based on the findings from our previous pilot study [25], comparing dynamic clearance between four stroke and four healthy participants, wherein the effect size was estimated to be between 0.4 (diagonal obstacle approach) and 0.5 (head-on obstacle approach). Using this estimated effect size, a sample size of 6–9 participants per group was required for a power of 0.80 and type-I error of 0.05. However, additional participants were included to ensure adequate power and minimal type-I error. The four post-stroke and four healthy participants from the previous pilot study [25] were also included in the present study that employed a slightly different research protocol. Each participant signed an informed consent form on inclusion in the study. The project was approved by the Montréal Centre de Recherche Interdisciplinaire en Réadaptation (CRIR) research ethics board.

Inclusion criteria for the persons with stroke were: 1) first incident of supratentorial stroke at least 6 months previously and localized to the middle cerebral artery territory; 2) age between 40 and 70 years; 3) independent ambulation ≥30 m with or without a walking aid; 4) normal or corrected to normal vision; 5) intact cognitive function (>27 score on the Mini-Mental Scale Examination (MMSE), as reported in the medical charts); and 6) stage 3/7 or greater on the leg component score of the Chedoke-McMaster Stroke Assessment (CMSA). Participants with visual field defect (information obtained through medical charts), hemineglect (screened using the Bell’s cancellation test), and other co-morbidities that interfered with walking or motion sickness (information obtained from patient interview) were excluded.

### Clinical assessment

Clinical assessments included comfortable gait speed (CGS; 10 m walk test [28]), motor ability (leg and foot scores of the CMSA [29]), balance confidence (Activities specific Balance Confidence Scale (ABC) [30, 31]), executive function and cognition (Montreal Cognitive Assessment Scale (MoCA) [32]), visuo-spatial perception, set-switching and working memory (Trail-Making A and B [33]) and handedness (Edinburgh Handedness Inventory [34]). The MoCA scale was used as a clinical assessment after screening participants with the MMSE scores (obtained through the charts) to determine the current cognitive status.

### Virtual environment

The virtual environment (VE) consisted of a 11 m × 8 m virtual room, including a central target and three red cylindrical obstacles arranged around an arc of radius 4 m centered at the theoretical point of intersection (PoI) [25]. The PoI is the central location where a moving obstacle crosses the midline and where a virtual collision would have occurred (with the walking subject moving at the same speed) if no avoidance strategy was implemented. The VE was projected using a helmet mounted display (Kaiser Optics; FOV: 50° diagonal; screen resolution: 1280 × 1024 pixels) and was created in the CAREN-3 (Computer Assisted Rehabilitation ENvironments, Motek Medical B.V.) software [25].

Locomotor and perceptuomotor tasks were evaluated in two separate sessions scheduled within a week. In both tasks, participants were instructed to navigate towards the target while avoiding a collision with the obstacle that was either stationary (at PoI), or randomly approaching from head-on, 30° left (L) or right (R) at a speed of 0.75 m/s, after the participants advanced 0.5 m in the VE.

### Locomotor task

Participants walked towards the target without any specific instructions on strategies except to avoid collision. Their position in the physical space was synchronized with that in the VE using a 12-camera Vicon-512 motion capture system (Vicon Motion Systems Ltd. UK) that relayed real-time positions of three reflective markers placed on the HMD to the CAREN-3 software. A collision event was detected when a surface-to-surface contact with the obstacle occurred at which point a placard flashing the word ‘collision’ appeared on the screen.

Two habituation blocks, i.e. walking without obstacles towards the target (5 trials) and walking in the presence of obstacles moving at the participants’ CGS (8 trials), were provided. The experimental blocks consisted of 6 trials per obstacle condition distributed randomly over 2 blocks of 12 trials each. The last block involved walking in the VE without obstacles (5 trials). Rest periods were provided as needed.

### Perceptuomotor task

Participants navigated to the target in the same VE while avoiding collisions with the obstacles using a joystick (Attack™ 3 Joystick, Logitech) as they were sitting with elbows resting on a table with adjustable height. In order to facilitate perception of shoulder width and self-motion in the VE, participants first navigated through a narrow tunnel (6 m long), the width of which matched the participants’ shoulders, before entering the virtual room. Deviation from the straight path resulted in a bump with the surrounding walls of the tunnel (informed via visual feedback) and consequent repetition of the trial.

Stroke participants used their non-paretic hand and healthy participants used their dominant hand to operate the joystick. Moving the joystick in the anterior or posterior (AP) direction respectively increased or decreased the navigation speed, while moving it in the left-right direction caused movement in the mediolateral (ML) plane. Without any joystick motion, the VE was perceived to move forward at 0.75 m/s in the AP direction leading to a collision. After a habituation block (12 trials), the perceptuomotor task was assessed with 10 trials of each obstacle condition randomly distributed over 4 blocks.

### Outcome measures

*Dynamic clearance (DC):* average clearance from the obstacle maintained along the entire avoidance strategy centered at the minimum distance, computed with the modified Shepard’s Inverse Distance Weighting method [25, 35, 36].

*Instantaneous distance at crossing (IDC):* the minimum instantaneous distance between the participant and the obstacle at the point of crossing (similar AP spatial positions of the participant and obstacle).

*Number of collisions:* expressed as % trials resulting in a collision, where surface-to-surface contact was detected as IDC < 0.3 m (distance between the center of mass of the participant and the obstacle, assuming shoulder width of ~0.4 m).

*Distance from the obstacle at the onset of strategy (distance at onset):* distance at onset was measured as the instantaneous distance between the participant and obstacle at the onset of the avoidance strategy (time at which the maximum increase/decrease of the angular velocity commenced in the first half of each trial).

*Preferred side of circumvention:* expressed as % trials in which participants veered towards the L/P or R/NP side following the onset of the avoidance strategy.

### Statistical analyses

The locomotor and perceptuomotor tasks were analyzed separately. Within each task, stationary vs. moving (head-on) and diagonal L/P vs. R/NP obstacle approach conditions were analyzed separately. For diagonal approaches, the NP approach in post-stroke participants was compared to the R approach in healthy participants and likewise for P vs L. The outcomes DC, IDC and distance at onset were compared using separate 2 × 2 repeated measures mixed models (with Bonferroni adjustments for multiple post-hoc comparisons) to detect differences between (stroke vs. healthy) and within group (stationary vs. head-on; L/P vs. R/NP). Independent samples unpaired t-tests were used to identify between group differences for distance at onset for the stationary and the head-on conditions for both tasks separately. Participants’ preference to circumvent to the L/P or R/NP side was tested separately for each group and obstacle condition using the non-parametric binomial test, assuming an equal proportion (0.5) of participants demonstrating preferential circumvention to the L/P or R/NP side in >50% trials. The difference in clinical assessments between the healthy and stroke group was performed using unpaired t-tests. An alpha-level of 0.05 for the unpaired t-tests and binomial tests and an adjusted alpha level of 0.05 for the mixed model analyses were accepted as significant. Descriptive statistics were used to evaluate the number of collisions. Statistical analyses were performed using the SPSS software (v20.0, IBM Corporation, NY, USA).

## Results

### Participants

Participants’ demographic information and clinical assessment are provided in Table 1. In comparison with the healthy individuals, stroke participants walked significantly slower (both overground and in the VE) and had significantly lower scores on the MoCA, ABC and Trail Making A tests (Table 1).

**Table 1:** Participant demographics and clinical assessments

	Age (years)	Time since stroke onset (years)	Side of lesion (R/L)	Gait speed (m/s)	Gait Speed in VE (m/s)	MoCA score (/30)	Trail making test (s)		CMSA (/7)	Cane use (+/−)	ABC (%)	Delay in detection of obstacle (s)		
							A	B	Leg,foot			Head-on	NP/R	P/L
*S1*	46	2.5	R	0.68	0.23	26	26.40	55.18	5,4	+	60.63	1.25	0.74	0.75
*S2*	54	4	R	0.31	0.26	23	47.80	65.50	3,2	+	71.25	2.39	0.68	0.85
*S3*	59	3	R	0.42	0.55	27	20.68	37.75	5,3	+	86.88	0.94	0.54	0.58
*S4*	60	2	L	0.36	0.2	19	37.79	172.00	3,3	+	50.94	0.70	0.53	0.59
*S5*	51	6	L	0.9	0.66	25	43.19	120.69	4,2	+	48.13	1.41	0.74	0.93
*S6*	54	2.5	R	1.27	0.67	29	28.89	45.06	5,4	−	83.13	0.78	0.54	0.46
*S7*	48	2	L	1.15	0.63	23	43.61	85.48	4,4	+	71.25	0.64	0.53	0.52
*S8*	52	2.75	L	1.36	1.04	25	28.10	49.00	7,6	−	90.31	0.55	0.46	0.46
*S9*	62	1	R	0.7	0.77	23	55.31	110.00	6,5	−	64.06	1.16	0.67	0.71
*S10*	66	7	R	1.3	0.93	26	28.91	83.00	6,5	−	89.38	1.31	0.84	0.81
*S11*	51	5	L	1.09	0.71	22	138.0	210.15	5,4	+	48.75	2.75	2.31	2.11
*S12*	68	1.5	L	0.73	0.46	21	86.00	296.00	5,4	−	67.50	1.36	0.64	0.71
*Stroke* *(Mean(SD))*	56.0 (7.0)	3.3 (1.9)		0.86 (0.38)*	0.59 (0.27)	24.08 (2.79)*	48.72 (33.13)*	100.18 (60.38)			69.34 (15.57)*	1.27 (0.68)	0.77 (0.50)	0.79 (0.44)
*Control* *(Mean(SD))*	52.5 (8.3)	−	−	1.49 (0.21)	0.98 (0.14)	28.25 (1.29)	24.57 (5.32)	61.65 (25.00)	−	−	94.47 (5.95)	0.84 (0.14)	0.64 (0.13)	0.63 (0.12)

Included within the table are results from an obstacle motion detection task, where participants were asked to press a button on a joystick (Attack™ 3, Logitech) as soon as they detected motion of one of the obstacles that approached from head-on, 30° left or right. VE: virtual environment, MoCA: Montreal Cognitive Assessment, CMSA: Chedoke-McMaster Stroke Assessment scale, ABC: Activities-Specific Balance Confidence scale, NP: non-paretic, P: paretic, R: right, L:left. *Unpaired t-tests, *p* < 0.05.

### Stationary vs. moving obstacle

No collisions were recorded in healthy participants for either the locomotor or perceptuomotor task and in the stroke participants for the perceptuomotor task. During walking, one stroke participant had one collision with the stationary obstacle, while three stroke participants had one collision each with the moving obstacle.

**Fig. 1.**
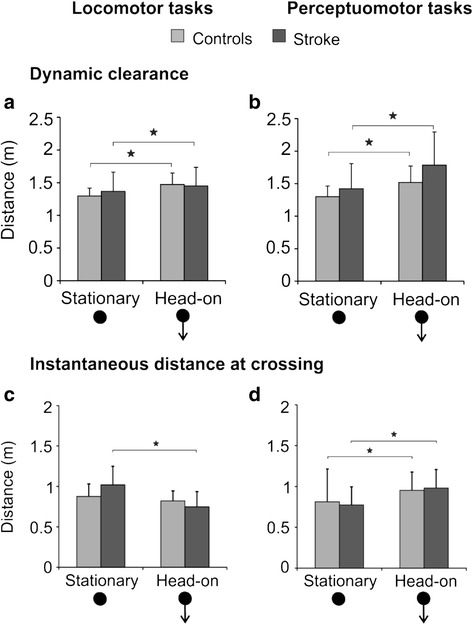
Dynamic clearance (DC) and instantaneous distance (IDC) for stationary and head-on approaches. Comparisons are shown between the stroke (*dark shade*) and control groups (*light shade*): DC mean ± SD for (**a**) locomotor and (**b**) perceptuomotor tasks; IDC mean ± SD for (**c**) locomotor and (**d**) perceptuomotor tasks. *p < 0.05. Black arrows following the black spheres at the bottom of the graph indicate direction of obstacle approach.

In both locomotor and perceptuomotor tasks, significant main effects due to obstacle characteristics were observed (locomotor: df = 22, F = 10.908, *p* < 0.01; perceptuomotor: df = 22, F = 79.611, *p* < 0.001; Fig. 1a & b) wherein both stroke and healthy participants maintained a larger DC for the moving obstacle as compared to the stationary. No significant main or interaction effects due to group or obstacle x group, respectively, were found.

For the locomotor task, a significant interaction effect (df = 22, F = 7.412, *p* < 0.05) of group x obstacle approach was found (Fig. 1c & d), wherein IDC was significantly smaller for the moving obstacle as compared to the stationary obstacle in stroke participants only (*p* < 0.001), while no difference was observed in healthy participants. For the perceptuomotor task, a significant main effect (df = 22, F = 24.259, *p* < 0.001) due to obstacle condition was found wherein IDC was larger for the moving obstacle in both groups (Fig. 1d). However, as opposed to the locomotor task, no significant effects due to group or group x obstacle were found.

As the moving (8 m) and stationary (4 m) obstacles were placed at different initial positions from the participant, only group differences for each obstacle condition and task were analyzed. However, distance from the obstacle at the onset of the circumvention strategy was not significantly different between the stroke and healthy participants regardless of the task and obstacle condition.

**Fig. 2:**
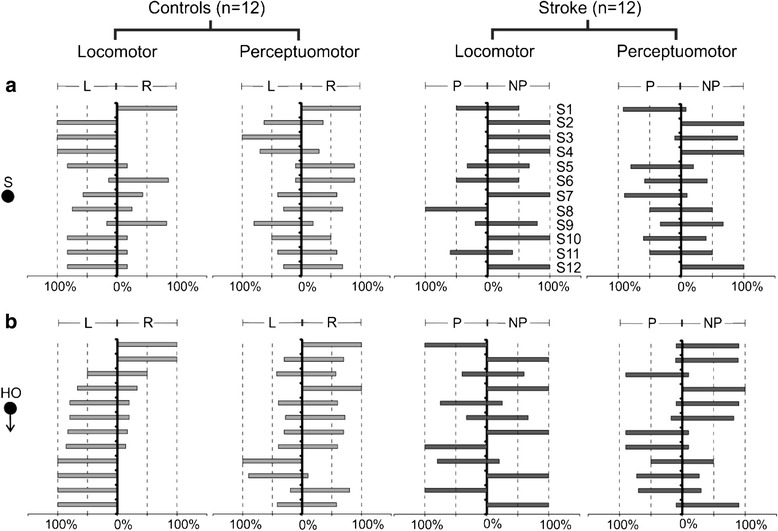
Preferred side of circumvention (% trials) in locomotor & perceptuomotor tasks for stationary and head-on approaches. Comparisons are made between stroke and control participants in their preferred circumvention (either towards the paretic/left and non-paretic/right side) for (**a**) stationary obstacle (S) and (**b**) head-on (HO) obstacle approach. S1-S12 denote post-stroke participants as described in Table 1. Participants are represented in the same order in all the panels. Black arrows following the black spheres at the side of the graph indicate direction of obstacle approach

In the locomotor task, 75% of healthy participants (9/12 for stationary (Fig. 2a) and 9/12 for moving obstacles (Fig. 2b) circumvented towards the left in >50% trials. This proportion was slightly larger than the expected 50% wherein an equal number of participants were expected to veer towards either side. However, this difference was not statistically significant (*p* = 0.15). In the stroke group, a larger number of participants circumvented to the NP side in >50% trials during walking (10/12 for stationary obstacle (*p* = 0.04); 7/12 for moving obstacle (*p* = 0.39); Fig. 2a & b). During joystick navigation, more healthy participants circumvented to the right side (8/12 for stationary (*p* = 0.39); 10/12 for head-on (*p* = 0.04)), and more stroke participants circumvented towards the non-paretic side (8/12 participants (*p* = 0.39) for both stationary and moving obstacle; Fig. 2a & b).

### Paretic vs. non-paretic obstacle approaches

No collisions were observed among the healthy participants in either tasks and among stroke participants in the joystick navigation task. During walking, two stroke participants (participants S11 and S12, see Table 1) collided with the *paretic-sided obstacle* approach in 20% and 60% trials respectively, while three stroke participants (S11, S12, S10) collided with the *non-paretic sided* obstacle approach in 75%, 25% and 16.67% trials respectively.

**Fig. 3:**
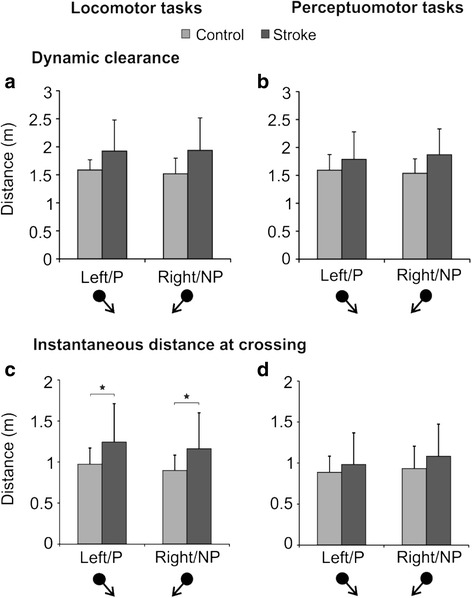
Dynamic clearance (DC) and instantaneous distance (IDC) for diagonal obstacle approaches. Comparisons are shown between the stroke (dark shade) and control groups (light shade): DC mean ± SD for (**a**) locomotor and (**b**) perceptuomotor tasks; IDC mean ± SD for (**c**) locomotor and (**d**) perceptuomotor tasks. R/NP: right/non-paretic, L/P:left/paretic. *p < 0.05. Black arrows following the black spheres at the bottom of the graph indicate direction of obstacle approach

No significant main or interaction effects were found when DC was compared across groups and obstacle approach directions in the locomotor and perceptuomotor tasks (Fig. 3a & b). However, for IDC, a significant main effect (df = 22, F = 4.447, *p* < 0.05) due to group was observed in the locomotor task, as the stroke participants maintained larger IDC than the healthy participants for both paretic and non-paretic approaches (Fig. 3c). In the perceptuomotor task, no significant main or interaction effect was found (Fig. 3d).

No significant main or interaction effects were found when distance at onset was compared across groups and obstacle approach directions in both tasks, indicating that both groups initiated avoidance strategies at similar distances from the obstacle, regardless of their direction of approach.

**Fig. 4:**
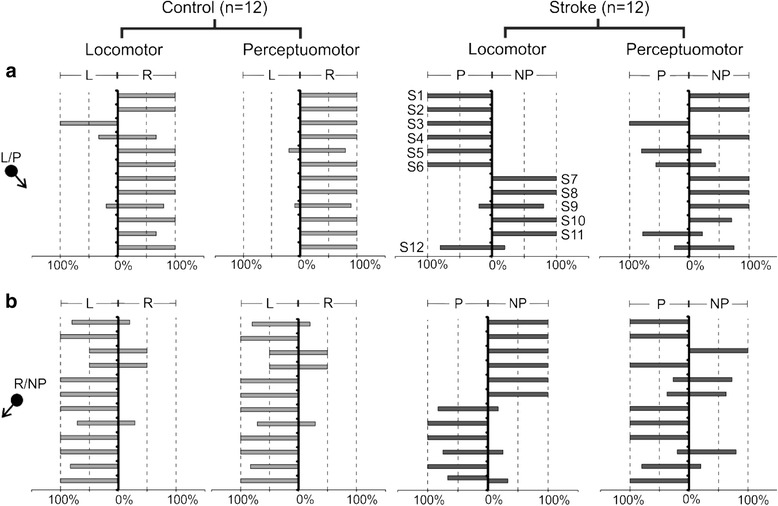
Preferred side of circumvention (% trials) in locomotor & perceptuomotor tasks for diagonal obstacle approaches. Comparisons are made between stroke and control participants in their preferred circumvention for obstacle approaching from (**a**) left/paretic (L/P) side and (**b**) right/ non-paretic side (R/NP) side. S1-S12 denote post-stroke participants as described in Table 1. Participants are represented in the same order in all the panels. Black arrows following the black spheres at the side of the graph indicate direction of obstacle approach

In both the locomotor and perceptuomotor tasks, healthy participants veered opposite to the direction of obstacle approach in >50% trials [locomotor: left obstacle approach–11/12 towards the right (*p* = 0.01), right obstacle approach–10/12 towards the left (*p* = 0.04); perceptuomotor: left obstacle approach–12/12 towards the right (*p* = 0.00), right obstacle approach – 11/12 towards the left (*p* = 0.01) (Fig. 4a & b)]. Although a larger number of stroke participants (8/12) veered opposite to the obstacle approach in the perceptuomotor task, the preference was not significantly different (*p* = 0.39). There was no significant preference of veering direction for the locomotor task, with ~50% (6/12 for the paretic approach and 7/12 for the non-paretic approach, *p* = 1.00) veering to either side of the obstacle approach (Fig. 4a & b). The stroke group thus behaved differently based on the preferred side of circumvention, circumventing to the opposite side like the control group (V_opp_: S7-S10, Table 1), or to the same side (V_same_: S1-S6). The V_same_ sub-group walked at lower gait speeds both in the VE and overground, had affected motor control, lower balance confidence and most (5/6) used a walking aid as compared to the V_opp_ sub-group. This sub-group also maintained larger DC (P: 2.14 ± 0.38 m; NP: 2.27 ± 0.37 m) than the V_opp_ sub-group (P: 1.58 ± 0.39 m; NP: 1.40 ± 0.34 m) for both obstacle directions.

## Discussion

This study is the first to demonstrate that stroke alters avoidance strategies while walking, depending on the obstacle contexts (greater clearance for moving obstacles vs. stationary) and the functional restrictions of the stroke individuals (different preference for side of circumvention). Moreover, similar avoidance behaviors in the perceptuomotor task but not in the walking task between healthy and post-stroke individuals, suggests that obstacle circumvention strategies in chronic stroke survivors in this study were largely shaped by their residual motor capacity. Although the present study included participants from a previous pilot study [25], it should be noted that they followed slightly different experimental procedures. Whereas in the previous study, the obstacle speed varied with the participants’ comfortable walking speed, the present study used a constant obstacle speed of 0.75 m/s. In addition, the present study included a stationary obstacle condition that was not used in the previous study.

### Stationary Vs. Mobile obstacle

Both healthy and post-stroke participants maintained larger DC from the moving obstacle as compared to the stationary, suggesting that participants may have opted for a cautious avoidance strategy for safe clearance from an approaching obstacle. This finding is in contrast to a previous study [19] showing a decrease in personal space when circumventing a moving obstacle as compared to a stationary one. Both outcomes, personal space and DC, are calculated differently (while personal space is an elliptical area, DC is the average weighted distance from the obstacle). In addition, difference in experimental paradigms, such as slower obstacle speed and head-on approach direction used in our study, both of which could invoke larger path adjustments and clearance [27], may have also contributed to the different results.

**Fig. 5:**
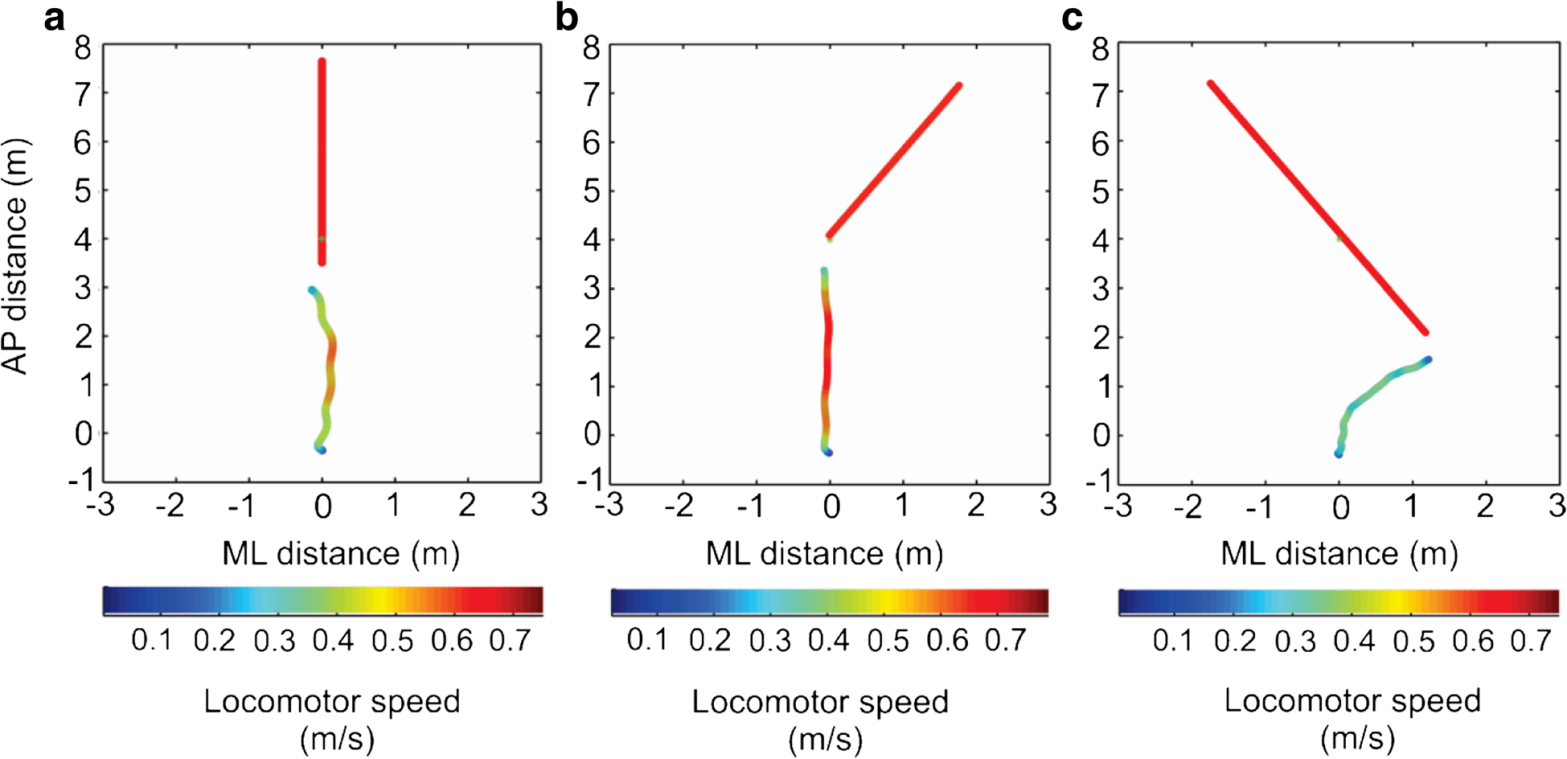
Collision trials. Examples of collisions that occur during different obstacle approaches in post-stroke participants: (**a**) head-on (S5), (**b**) paretic (S11) and (**c**) non-paretic (S12). The trajectories are colour-coded to represent locomotor speed in m/s. Colour bars at the bottom of each figure provide the colour coding and range of velocities

In contrast to DC, IDC was similar for both stationary and moving obstacles in healthy participants, as reported previously [19], indicating that distance from the obstacle is maintained fairly constant when one is closer to the obstacle. Conversely, stroke participants maintained a smaller IDC for moving obstacles as compared to stationary, suggesting that despite an attempt to adopt cautious strategies, the safe margin from the obstacle may have shrunk for this group, possibly due to added processing demands imposed by the moving obstacle. This may also explain a slightly larger likelihood for collision with obstacles approaching head-on in stroke participants (Fig. 5a). Although these findings agree with our hypothesis that individuals with stroke use cautious strategies for avoidance overall, a smaller clearance at obstacle crossing may pose some threat of collision especially with moving obstacles.

### Diagonal obstacle approaches

IDC was greater in stroke participants than healthy while DC showed a similar trend when obstacles approached diagonally, suggesting that as compared to healthy individuals, post-stroke individuals employ cautious strategies for circumvention around diagonally approaching obstacles as well, especially in the locomotor task.

The two sub-groups based on the difference in preferred side of circumvention (V_same_ and V_opp_) in response to diagonally moving obstacles differed from each other in functional abilities, indicating that functional limitations may influence avoidance strategies in individuals with stroke. The V_same_ sub-group that had greater functional limitations may have chosen to circumvent to the same side as the obstacle approach as a cautionary measure to decrease the risk of collision. Circumvention to the opposite side of obstacle approach places an individual in the path of the obstacle, thus increasing collision risk and requiring the individual to walk faster than the obstacle to pass in front of it to successfully avoid a collision. Greater functional restrictions seen in the V_same_ sub-group may have precluded them from generating large changes in gait speed [37, 38], thus significantly increasing collision risks with a front pass. A relatively risk-free choice of circumventing to the same side of obstacle approach with a larger DC may thus have been employed, indicating that post-stroke individuals with more functional restrictions may choose cautious strategies for obstacle circumvention.

Further, the difference in avoidance strategies seen amongst the post-stroke participants may have resulted from the interaction of their functional abilities rather than differences in gait speed alone. For instance, if gait speed were the sole influential factor shaping avoidance, participants with similar gait speeds while walking in the VE (for instance participants S5 (0.66 m/s) and S7 (0.63 m/s); Table 1) would have demonstrated similar avoidance strategies. However, despite walking at similar speeds, participants demonstrated contrasting strategies, as S5 circumvented to the same side as the obstacle approach while S7 circumvented to the opposite side. These strategies may have been influenced by factors related to functional capacity, as S5 had greater restrictions of motor ability (CMSA stage of foot impairment – S5: 2, S7:4) and lower balance confidence (ABC – S5: 48.13%, S7: 85.48%). These may have resulted in an inability to increase the walking speed beyond that of the obstacle and may have consequently led to a cautious avoidance strategy adopted by S5. Thus a combination of factors may have led to differences in avoidance strategies seen in the stroke group in this study.

Two post-stroke participants (S11, S12, Table 1) demonstrated higher rates of collisions with the paretic and the non-paretic sided obstacle approaches respectively. Participant S11, who collided with the obstacle approaching from the paretic side, failed to generate timely, adequate veering (Fig. 5b), due to possible difficulties in making appropriate segmental rotations and/or stepping adjustments [39], as well as cognitive-perceptual deficits. Further investigation of the perceptuomotor task revealed that participant S11 maintained smaller dynamic clearance for paretic-sided obstacle approach (1.20 ± 0.54 m) than the non-paretic approach (1.63 ± 0.73 m) and demonstrated greater delays in an obstacle motion detection task (Table 1). Thus a combination of motor, cognitive and perceptual deficits may have contributed to a greater collision rate in this participant.

Participant S12 showed a higher collision rate with the non-paretic obstacle approach. Unlike participant S11, this participant collided with the obstacle while veering towards the opposite side of obstacle approach (Fig. 5c). However, this strategy was not successful as S12 walked at speeds far below the obstacle speed in the VE (0.46 m/s) and thus often collided with the obstacle. Although both participants had collisions, the causes may differ for each participant and are important to analyze to design interventions for collision prevention.

### Perceptuomotor task

The perceptuomotor task used in this study was included to explore cognitive-perceptual contributions to the obstacle circumvention task, in the absence of biomechanical constraints imposed by the hemiparetic lower extremity during the locomotor task. The joystick navigation task has been previously used in the post-stroke population with and without visuospatial neglect [40, 41]. The outcomes DC and IDC obtained from the perceptuomotor task were not compared with the locomotor task as the experimental paradigms were slightly different (unlike the walking task, participants had to traverse a 6 m long tunnel while navigating the joystick). Additionally, it is known that differences in spatial outcomes exist between walking and joystick navigation tasks performed in virtual environments, wherein the displacements in the horizontal plane during navigation were found to be greater during the joystick navigation task as compared to walking [42]. Therefore, the circumvention strategies used during joystick navigation and walking were compared only qualitatively to explore similarities and/or differences in avoidance strategies.

Similar to the locomotor task, a larger number of individuals with stroke adopted cautious strategies when dealing with stationary and head-on approaching obstacles by preferentially circumventing towards the non-paretic side. However, increased caution was adopted by both healthy and post-stroke participants when encountered with a moving obstacle as both groups maintained larger DC when the obstacle was approaching head-on as compared to remaining stationary. Thus, the increased processing demands imposed by the head-on approaching obstacle appear to influence circumvention strategies in the perceptuomotor task as well.

A larger number of individuals with stroke also demonstrated similar strategies as healthy participants when avoiding obstacles that approached diagonally by circumventing towards the opposite side of obstacle approach. This was in contrast with the locomotor task where two distinct circumvention strategies were observed. These results suggest that when constraints imposed by the hemiparetic lower extremity on walking are minimized, circumvention strategies may be similar between individuals with stroke and healthy individuals. In a previous study in post-stroke individuals with visuospatial neglect, individuals with stroke demonstrated altered circumvention strategies in both the locomotor and perceptuomotor tasks leading to collisions [24, 41]. The perceptuomotor task in this study was thus indicative of deficits in the visuospatial perception that possibly lead to altered circumvention strategies in the locomotor task as well. The present study however, did not reveal any altered strategies in the perceptuomotor task indicating that in individuals without visuo-perceptual deficits, the perceptuomotor task may have limited utility and that altered circumvention strategies may be revealed through walking tasks.

Individuals with stroke included in our study used the unaffected hand to operate the joystick for navigation. It may be argued that circumvention strategies may have been affected due to coordination deficits on the unaffected side. While it is true that the unaffected upper limb in the stroke population may demonstrate motor and coordination deficits [43–45], it may have had limited effect in the present study as these deficits tend to recover with time [46, 47]. Also, circumvention strategies used by individuals with stroke in our study were similar to that used by healthy participants and dynamic clearance from the obstacle was also not significantly different between the two groups.

### Limitations

The present study found that circumvention strategies in people with stroke may differ based on their functional abilities. However, to what extent do these abilities affect circumvention behaviors has not been investigated. Also, the effect of each factor such as walking speed, balance confidence or motor ability on circumvention behaviors was not evaluated due to the variability in circumvention behaviors exhibited by the stroke participants and the relatively smaller sample size. The obstacle speed in the present study was held constant at 0.75 m/s to ensure common stimuli for all participants. This fixed obstacle speed in relation to the variable walking speeds of the participants may have introduced a source of variability. Future studies should explore the effect of obstacle and participant speeds on obstacle circumvention strategies. Further, this study did not evaluate the effect of walking speeds on circumvention strategies. It is yet to be investigated whether constraining walking speeds of the participants with stroke who walked faster would lead to a circumvention strategy used by the slower walking participants.

## Conclusion

This study of locomotor circumvention strategies showed that avoidance strategies used by post-stroke individuals may be dependent upon obstacle contexts and their functional abilities. Increased clearance from obstacles and circumventing to the same side of obstacle approach are indicative of a cautious strategy, when encountered with moving obstacles, particularly by individuals with greater functional restrictions. In addition, these individuals may be at risk of collisions; however, the underlying mechanisms resulting in collisions may vary. Assessment of locomotor obstacle circumvention and the subsequent interventions should therefore give due consideration to the individual functional capabilities, while incorporating appropriate obstacle characteristics such as the use of moving obstacles with varying approach directions and speeds.

## Abbreviations

ABC: Activities-specific Balance Confidence scale
AP: antero-posterior
CMSA: Chedoke McMaster Stroke Assessment
DC: Dynamic clearance
IDC: Instantaneous distance from obstacle at crossing
L: Left
ML: Mediolateral
MMSE: Mini Mental Scale Examination
MoCA: Montreal Cognitive Assessment scale
NP: Non-paretic
P: Paretic
PoI: Theoretical Point of Intersection
R: Right
VE: Virtual environment
